# Language Experience Modulates the Visual N200 Response for Disyllabic Chinese Words: An Event-Related Potential Study

**DOI:** 10.3390/brainsci13091321

**Published:** 2023-09-14

**Authors:** Jiang Liu, Yang Zhang

**Affiliations:** 1 Department of Languages, Literatures and Cultures, Linguistics Program, University of South Carolina, Columbia, SC 29208, USA; 2 Department of Speech-Language-Hearing Sciences & Masonic Institute for the Developing Brain, University of Minnesota, Minneapolis, MN 55455, USA

**Keywords:** ERP, N200, Chinese orthography, Korean Hangul, language experience

## Abstract

Prior event-related potential (ERP) research on how the brain processes non-alphabetic scripts like Chinese has identified an N200 component related to early visual processing of Chinese disyllabic words. An enhanced N200 response was observed when similar prime-target pairs were presented, but it was not elicited when native Chinese speakers read Korean Hangul, a script resembling Chinese characters. This led to the proposal that N200 was not a universal marker for orthographic processing but rather specific and unique to Chinese. However, there was uncertainty due to the absence of Korean participants in the previous research. The impact of language experience on N200 remains unclear. To address this, the present pilot ERP study included three adult groups (totaling 30 participants) with varying language proficiency levels. The participants judged if randomly presented words were Chinese or Korean, while the ERP responses were recorded. The behavioral data showed high accuracy across the groups. The reaction times differed between the groups with the native speakers responding faster. The N200 patterns varied across the groups. Both Chinese native speakers and Chinese-as-second-language learners showed stronger N200 responses for Chinese words compared to Korean words regardless of whether an adaptive or a fixed-time window was used for the N200 quantification, but this was not the case for Korean native speakers. Our cross-linguistic study suggests that N200 is not exclusive to Chinese orthography. Instead, it reflects general visual processing sensitive to both orthographic features and learning experience.

## 1. Introduction

Visual word reading is a multifaceted cognitive process that encompasses orthographic, phonological, and semantic stages [1,2,3,4,5]. Proficiency in these areas predicts reading fluency across various writing systems [6,7]. To study the temporal dynamics of word reading, early research employed a priming paradigm, manipulating the time gap (stimulus onset asynchrony, SOA) between the prime and target words. This approach helped to identify when orthographic, phonological, and semantic priming effects were initiated. Researchers determined the shortest SOA that induced priming effects, shedding light on the activation timeline of different information sources. This extensive behavioral research led to the proposal of two cognitive models. In alphabetic languages like English, orthographic input activates the mental lexicon and grapheme–phoneme mapping via dual pathways, concurrently triggering phonological and semantic activation through phonological similarity in the mental lexicon [8,9]. In contrast, non-alphabetic languages like Chinese that lack phonetic transparency tend to employ a threshold-style processing with phonological and semantic representations activated only after an initial orthographic analysis reaches a predefined threshold [10,11].

As the research field advances, electroencephalography (EEG) has been increasingly used to investigate the temporal dynamics of word reading. EEG records the electric potential signals from electrodes placed on the scalp, and event-related potentials (ERPs) are derived from averaging EEG epochs time-locked to stimulus presentation [12,13]. This approach enables a more direct examination of the temporal dimension of online cognitive processing such as word reading before an overt response is made. Previous ERP studies have pinpointed several neural markers for visual word reading. Among these, one of the earliest is the left-lateralized N170 response, which manifests as a negative peak at approximately 170 ms following stimulus onset [14,15,16]. This N170 response effectively distinguishes orthographic stimuli like words and pseudowords from other stimuli such as symbols or objects, and it is considered an automatic response associated with typical visual word recognition. An even earlier ERP component, N150, has also been found to react to the frequency of letter combinations in alphabetic language like English [17,18]. Another early marker of reading, the N250 or N2 component, typically occurs at approximately 200 to 350 milliseconds after stimulus onset. It is thought to reflect processes related to word form and recognition. The amplitude of the N250 can be modulated by factors such as word frequency and word familiarity, encompassing both orthography and phonological similarities [19,20]. Finally, the N400, typically identified between 350 and 450 ms from stimulus onset, is highly responsive to semantic information. Its sensitivity can be modulated based on the degree of semantic and contextual compatibility [21,22,23].

ERP research has led to a number of important discoveries on the cross-linguistic differences in word reading. For instance, perceptual expertise with fonts and letters has a notable impact on ERP components such as the N170, which play a crucial role in early visual analysis of (non)orthographic stimuli. Wong et al. conducted an ERP study where English monolinguals and Chinese–English bilinguals were asked to perform a one-back stimulus identity judgment on Roman letters, a Chinese monosyllabic word, and pseudo-fonts [24]. The Chinese–English bilingual participants exhibited an enhanced N170 response for both Roman letters and Chinese characters compared to pseudo-fonts. Conversely, for the non-Chinese readers, the N170 amplitude was greater for Roman letters when compared to Chinese characters and pseudo-fonts. These findings indicate that expertise with written language is closely tied to the strength of the N170 response.

The Chinese logographic (also referred to as morphosyllabic) writing system is fascinating to many researchers as it is structurally distinct from thousands of alphabetic or phonologically-based writing systems and can be very challenging to learn (e.g., 国 ‘nation’, 汽 ‘gas’) [25,26]. Although Chinese script has monosyllabic words, the majority of modern Chinese words are disyllabic (monosyllabic words: 2.8%; disyllabic words: 63.9%) [27]. Early ERP studies on Chinese visual word recognition used single-character stimuli. However, these monosyllabic characters cannot represent word processing for typical disyllabic Chinese words, which are two square-shaped blocks next to each other (e.g., 国家 ‘nation’, 汽车 ‘automobile’). One hot topic for ERP researchers is to investigate whether there exists some ERP component that is uniquely related to logographic writing systems.

While the N170 has been consistently observed in ERP studies across both alphabetic and non-alphabetic languages, the same cannot be said for the N200. Using disyllabic Chinese words as stimuli, a few early studies found the early ERP component N200 (a negative peak at approximately 200–250 ms after the onset of the stimulus) instead of N170 in a delayed naming task [28,29]. Because these studies did not separate orthographic and phonological processing within the task, it remained unclear whether the N200 was linked to early orthographic processing of disyllabic Chinese words. To address this issue, Zhang and associates [1,30] were the first to conduct a series of experiments to determine whether the centro-parietal N200 was uniquely related to Chinese orthography in the context of processing disyllabic words. One important finding was the elicitation of N200 specifically by Chinese words but not by alphabetic scripts. Zhang et al. [1] showed that there was a clear and enhanced N200 response upon repetition of Chinese orthography, whereas phonological and semantic priming did not appear to affect N200. It was thus proposed that the N200 reflected prelexical orthographic processing for reading the two-character Chinese words. This component is considered independent of the earlier ERP component known as the N170 that was found to be responsible for the initial visual analysis of a stimulus [16,31,32]. To verify whether this N200 is uniquely related to processing Chinese orthography, Zhang et al. [1] chose the Korean alphabet, Hangul, for comparison. Hangul script (e.g., 명부) is similar to Chinese characters in terms of the visual square-shape composition and orientation of the parts in a writing unit, but it maps letters/graphemes onto phonemes like English does. The results showed that the N200 was present for the disyllabic Chinese words but not the Korean words, which indicates that the N200 response reflects deep word-form processing specifically for Chinese orthography. However, it is worth noting that the Chinese participants’ lack of Korean language knowledge may have preemptively voided deep orthographic processing of the Korean script.

There is substantial evidence indicating that one’s learning experience with a particular writing system significantly impacts ERP responses. In addition to the previously mentioned study by Wong et al. [24], which showed how expertise with print influenced N170 responses during orthographic processing, several research groups have further demonstrated that enhanced visual familiarity or expertise in learning Chinese leads to a pronounced left-lateralized N170 response for Chinese characters [33,34,35,36,37]. Similar to the N170 response, Liu, Perfetti, and Wang [29] found that native English speakers who had some experience learning Chinese showed a larger N200 response for disyllabic Chinese words than for English words. Recent studies have also demonstrated that visual word recognition is not solely a product of cognitive processing within the visual modality. While font style affects the N170 but not the N200 response for reading Chinese words [38], the N200 response is influenced by motor-gesture decoding, which reflects stroke orientation and sequential execution in handwriting that develops over years of practice [39,40].

To date, there has not been a systematic study to examine how varying experience with Chinese and Korean languages may differentially affect the N200 response. To address this knowledge gap, we recruited three groups of participants with varying levels of Chinese and Korean language experience and used a simple task to minimize the influence of phonological and semantic processing. We compared the behavioral accuracy and reaction time data with a simple hypothesis that the behavioral measures would demonstrate an advantage in favor of the two native speaker groups over the L2-Chinese learners who did not have the same level of reading expertise. For the ERP measure, we tested two main hypotheses: the first was that the Chinese learning experience would lead to a deeper level of orthographic processing of Chinese characters, leading to an enhanced N200 for Chinese scripts relative to Korean scripts, and the second was that the native Korean speakers would also show a strong N200 when processing Korean Hangul, which may be equivalent to or even stronger than that for Chinese characters. As previous research has revealed both language-universal and language-specific reading mechanisms across different writing systems [41,42,43], the pilot cross-linguistic ERP study on visual word reading in Chinese and Korean scripts shall further shed light on the understanding of how learning experiences affect orthographical processing of logographic and syllabic writing systems.

## 2. Methods

### 2.1. Participants

The current study recruited 30 participants from a public university in the U.S., including 10 native Chinese speakers who had no formal learning experience with the Korean language (L1-Chinese group), 10 native Korean speakers who had no formal learning experience with the Chinese language (L1-Korean group), and 10 English-speaking L2-Chinese learners who had learned Chinese for at least 8 semesters and achieved an advanced proficiency level assessed with a standardized proficiency test administered by the American Council for Teaching Foreign Languages (ACTFL) (L2-Chinese group). The demographic information and language experience information are shown in Table 1. All participants had normal or corrected-to-normal vision, were right-handed, and reported no history of language or speech disorders. The L2-Chinese group had passed an advanced level Chinese proficiency test. Written informed consent was obtained from each participant in accordance with the University of Minnesota’s IRB protocol, and they were compensated for their participation.

### 2.2. Stimuli

Following Zhang et al. [1], the current study utilized disyllabic words as the stimuli, which consisted of 36 genuine Chinese words (e.g., 名单) and 36 real Korean words (e.g., 명부). Two Chinese and two Korean words were used for practice trials in addition to the target words. The Chinese words had an average frequency of 37.9, and the number of strokes was equivalent for the Chinese and Korean words (11.0 vs. 11.2, *p* > 0.05).

### 2.3. Procedure

A language script decision task was adopted in which the participants had to quickly and accurately determine whether the words displayed on a computer screen were Chinese or Korean. There were four testing blocks, each containing 74 words stimuli (including 2 practice words and 72 target words) with an equal number of Chinese and Korean words randomly mixed. Prior to each testing block, the participants completed a separate practice block with feedback that consisted of 6 trials with an equal number of Chinese and Korean words.

The participants were positioned 0.7 m away from the computer screen in a soundproof and electrically shielded room. They were instructed to keep their head still and avoid blinking during the EEG recording. Each trial began with a 500 ms fixation cross followed by the presentation of a word at the center of the screen for 400 ms. The participants were asked to identify whether the word was written in Chinese or Korean script and to respond by pressing ‘F’ for Chinese and ‘J’ for Korean within a 1900 ms window. The response speed and accuracy were recorded. There was a random blank interval of 600 to 800 ms between each response and the next fixation. While the participants completed the task, the experimenter monitored the ERP recordings in an adjacent room.

### 2.4. EEG Recording

**Figure 1:**
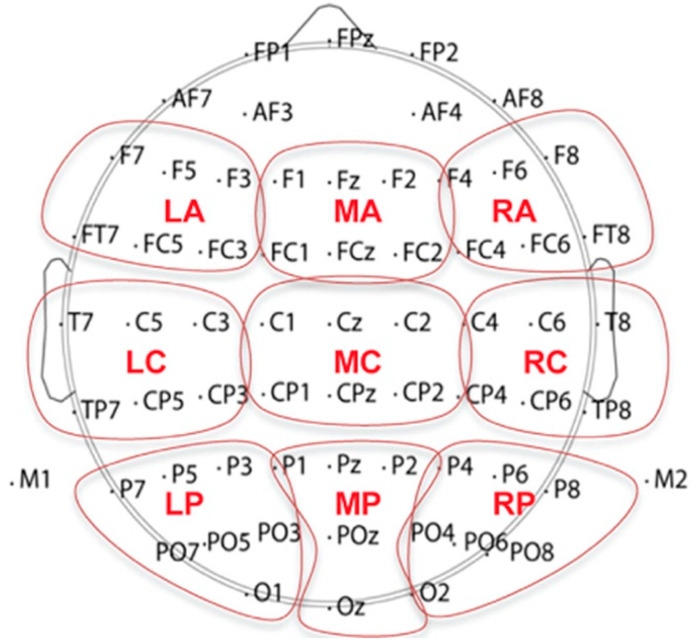
Layout of 64 electrodes and channel grouping of 9 electrode regions for ERP data analysis.

During the EEG recording, the participants sat on a comfortable chair inside a soundproof booth (ETS-Lindgren Acoustic Systems). They wore a stretchable, 64-channel Waveguard cap, and their continuous EEG data were recorded using the Advanced Neuro Technology system with a bandwidth of 0.016–200 Hz and a sampling rate of 512 Hz. The Ag/AgCl electrodes were positioned based on the standard International 10–20 Montage System and intermediate locations with the ground electrode located at the AFz electrode (as shown in Figure 1). To ensure a smooth fitting procedure, each electrode was surrounded by a silicone ring that held the conductive gel. Prior to the EEG recording, the impedance of each electrode was adjusted to maintain levels at or below 5 kΩ.

### 2.5. ERP Analysis

ERP analysis was performed in MATLAB (Version 9.10) with the EEGLAB software (Version 2021.1) [44]. A bandpass filter of 0.5–40 Hz was used. The ERP epoch length was 900 ms, including a pre-stimulus baseline of 100 ms. Independent component analysis was conducted to help reduce the artifacts from eye blinks and other artifacts following the guideline by Chaumon et al. [45]. The ERP data were then re-referenced to the average of the left and right mastoids. Trials with potentials beyond the range of plus or minus 50 μV were automatically rejected. Following previous studies, nine electrode regions were used for averaging the ERP responses for each participant for the statistical analysis (see Figure 1). They were named left frontal (LF, including F7, F5, F3, FT7, FC5, and FC3), middle frontal (MF, including F1, Fz, F2, FC1, FCz, and FC2), right frontal (RF, including F8, F6, F4, FT8, FC6, and FC4), left central (LC, including T7, C5, C3, TP7, CP5, and CP3), middle central (MC, including C1, Cz, C2, CP1, CPz, and CP2), right central (RC, including T8, C6, C4, TP8, CP6, and CP4), left posterior (LP, including P7, P5, P3, PO7, PO5, and PO3), middle posterior (MP, including P1, Pz, P2, and POz), and right posterior (RP, including P8, P6, P4, PO8, PO6, and PO4) [46,47,48].

The mean amplitudes of a 40 ms interval around the N200 peak were subjected to factorial ANOVA tests. The N200 peak search window was determined by examining the grand mean ERP overlay plots and topography as well as the ERP waveforms for each participant, which fell within 200–300 ms. The within-subject factors included the type of stimulus (Chinese and Korean script), hemisphere (left and right), and electrode region (frontal, central, and parietal), while the between-subject factor was the language group (L1-Chinese, L2-Chinese, and L1-Korean). In the case of multiple comparisons, the reported *p*-values were adjusted using either Bonferroni or Greenhouse-Geisser correction.

The quantification of the N200 amplitude used an adaptive flexible window around the individual N200 peak latency, which may not be sensitive to detect time-aligned subject group differences in the ERP waveforms between the Chinese and Korean script conditions [49]. To identify point-to-point differences between the ERPs for each language group, a two-tailed cluster-based permutation test was conducted using the ERP data from the Chinese and Korean script conditions. This test had a family-wise significance level of 0.05, and the time points from 0 to 400 ms (including the N200 window of 200–300 ms) were used with 5000 permutations applied to determine the significant clusters. The global field power (GFP) waveform, which captures the standard deviation of the ERP amplitude data points across all electrodes at each time point of the epoch window, was computed for this purpose. Using GFP data avoids potential biases in electrode selection and allows for the capture of global patterns of ERP differences on the time scale. Previous studies have shown that the permutation test method on GFP data is sensitive and valid for detecting within-subject differences, even with unbalanced ERP data [50].

## 3. Results

### 3.1. Behavioral Results

The language decision task was found to be easy, and all three language groups performed very well with an accuracy rate of 95% or higher for both Chinese and Korean scripts. When analyzing the response times, a main effect of language script was observed (F(1,8607) = 63.9, *p* < 0.001), indicating that the participants responded faster to Korean script compared to Chinese script (Korean script: 488 ms vs. Chinese script: 507 ms). There was also a significant interaction effect between language script and language group (F(2,8607) = 3.9, *p* < 0.05). Post-hoc analysis revealed that, for the Chinese script, both the L1-Chinese and L1-Korean groups had faster response times than the L2-Chinese group (*p* < 0.001), but no difference was found between the L1-Chinese and L1-Korean groups (Chinese script, L1-Chinese: 488 ms; L1-Korean: 494 ms; L2-Chinese: 536 ms). For the Korean script, both the L1-Chinese and L1-Korean groups responded faster than the L2-Chinese group (*p* < 0.001). Additionally, the L1-Korean group responded faster than the L1-Chinese group (Korean script, L1-Chinese: 478 ms; L1-Korean: 468 ms; L2-Chinese: 513 ms). These findings are consistent with our hypothesis: despite the ceiling-level performance in terms of accuracy across all three groups, a clear advantage in processing speed can still be observed in favor of native language expertise in the language script decision task.

### 3.2. ERP Results

**Figure 2:**
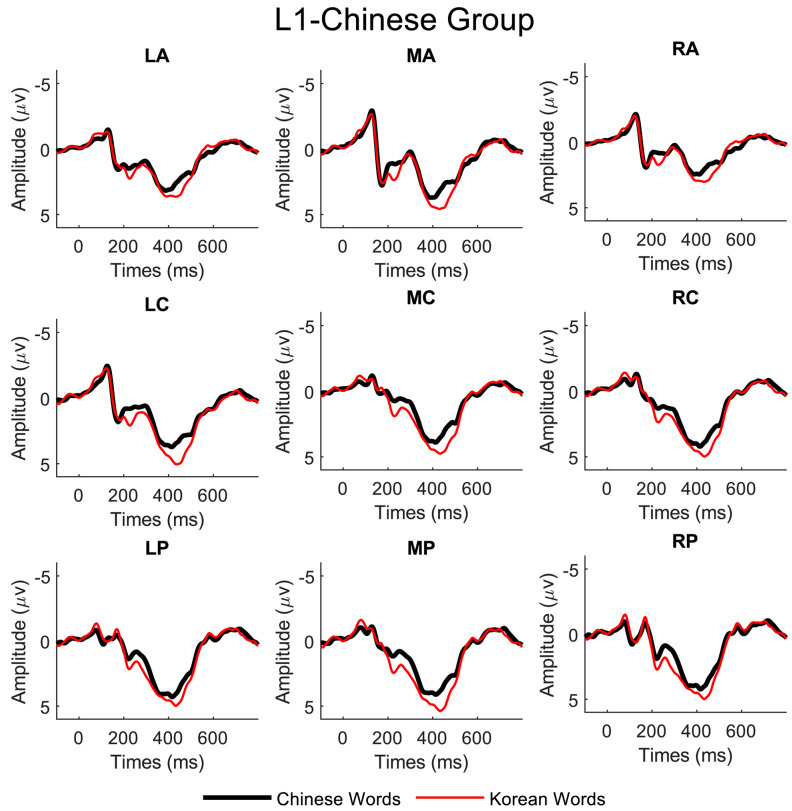
Grand average ERP data (average mastoids reference) of the nine electrode regions for the L1-Chinese group.

**Figure 3:**
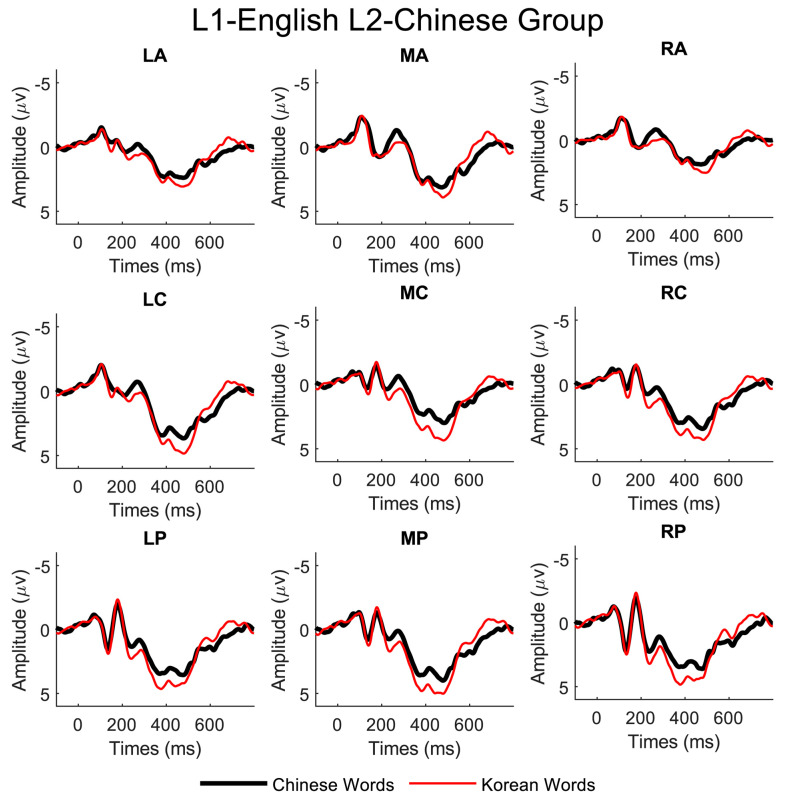
Grand average ERP data (linked mastoids reference) of the nine electrode regions for the L1-English L2-Chinese group.

**Figure 4:**
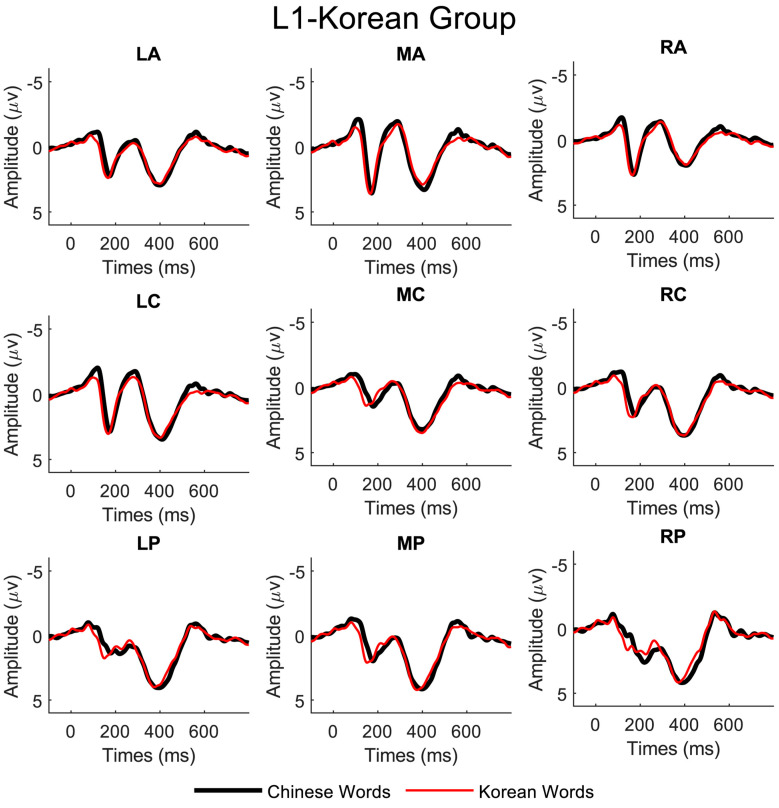
Grand average ERP data (linked mastoids reference) of the nine electrode regions for the L1-Korean group.

Distinct N200 peaks were observed between 200 and 300 ms after the words were presented on the screen for both Chinese and Korean scripts in all regions of interest for all three language groups. The ERP waveforms from representative electrodes are illustrated in Figure 2, Figure 3 and Figure 4.

We conducted repeated measures of ANOVAs to investigate the effects of three factors on the observed N200, namely language script (Chinese vs. Korean script), laterality (left, middle, and right), and site (anterior, central, and posterior), for the three subject groups (L1-Chinese, L2-Chinese, and L1-Korean). For the L1-Chinese group, we found significant main effects of language script (F(1, 9) = 17.98, *p* < 0.001) and laterality (F(2, 9) = 4.15, *p* < 0.05). For the L2-Chinese group, there were significant main effects of language script (F(1, 9) = 12, *p* < 0.001), laterality (F(2, 9) = 8.52, *p* < 0.001), and electrode site (F(2, 9) = 41, *p* < 0.001). For the L1-Korean group, we observed significant main effects of laterality (F(2, 9) = 14.9, *p* < 0.001) and site (F(2, 9) = 72, *p* < 0.001) but no significant main effect of language script.

**Figure 5:**
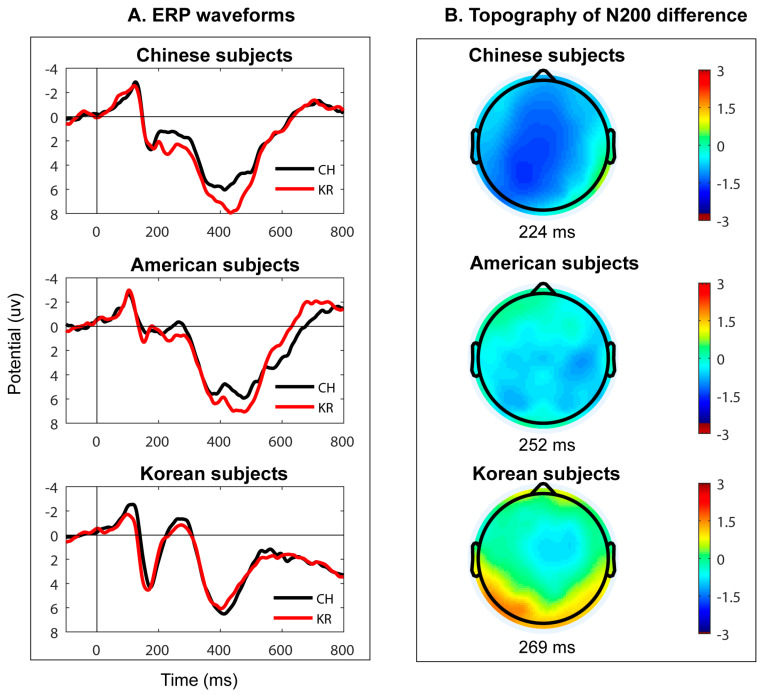
(**A**) Grand average ERP waveforms from the Cz electrode for the L1-Chinese (Chinese subjects), L1-English L2-Chinese (American subjects), and L1-Korean groups (Korean subjects) (CH stands for Chinese words, and KR stands for Korean Words). (**B**) Topography maps for peak N200 differences in ERP responses (Chinese script minus Korean script) for three subject groups.

To better understand the effects of language script, we compared the responses for Korean and Chinese script across all three subject groups by subtracting Korean script responses from Chinese script responses and plotting the N200 peak differences with topographical maps over the scalp in Figure 5.

**Figure 6:**
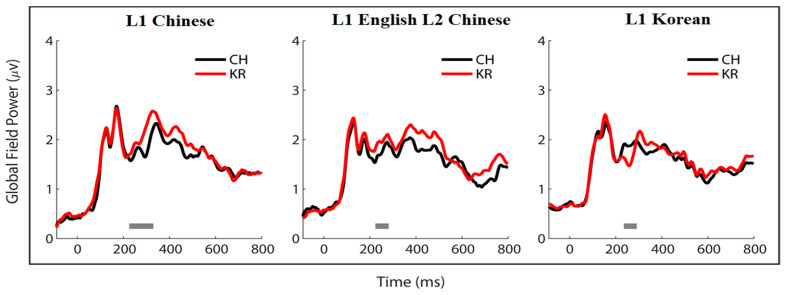
Cluster-based permutation test results for the within-subject global field power differences between Chinese and Korean scripts in the three subject groups. Significant time clusters (alpha level at 0.05) are marked as horizontal grey bars above the time scale.

The topographic maps clearly demonstrate strong effects of language experience with the L1-Chinese group showing the strongest N200 difference across all regions, the L2-Chinese group exhibiting a reduced N200 effect more localized towards the central and posterior regions, and the L1-Korean group displaying essentially no difference in most electrode regions except in the mid-central region. The peak latencies for the N200 difference waveforms were 224 ms for the L1-Chinese group, 252 ms for the L2-Chinese group, and 269 ms for the L1-Korean group. We performed cluster-based permutation tests on the GFP data and found significant point-to-point differences in N200 across the three subject groups, as shown in Figure 6. The L1-Chinese group had the earliest and longest significant time cluster from sample 174 to sample 214 (238–316 ms), while the L2-Chinese group had a shorter significant time cluster from samples 170 to 186 (230–262 ms). In contrast, the L1-Korean group showed a reversed amplitude trend compared to the other two groups with the significant time cluster extending from sample 176 to sample 191 (242–272 ms).

## 4. Discussion

The present study extended the work of Zhang et al. [1] by examining whether the N200 component of the ERP reflects the processing of word forms that is uniquely sensitive to Chinese orthography when compared to visually similar Korean Hangul. To accomplish this, this study utilized a simple language script decision task and included three groups of participants with varying levels of Chinese/Korean language experience. The results from both behavioral and ERP measures supported the idea that language experience has significant effects on how individuals process the two orthographic systems. Specifically, the ERP data showed that N200 reflects the visual processing of the spatial configuration of Chinese and Korean orthography, which is modulated by the language learning experience.

### 4.1. Reaction Time Shows a Disadvantage in the L2-Chinese Group

The behavioral results indicated that the L1-Chinese and L1-Korean groups responded significantly faster to Chinese script compared to the L2-Chinese group whose L1 was English. This could be due to the visual similarity between Chinese and Korean scripts as well as the L2-Chinese group’s relatively lower level of orthographic expertise, which may have slowed their responses in identifying the target words. The L1-Chinese and L1-Korean groups’ familiarity with their respective L1 orthography may have facilitated a quick decision. These data are consistent with previous research that showed slower lexical processing in bilingual individuals. Interestingly, our results showed an asymmetry in which the L1-Korean group responded faster to Korean script than the L1-Chinese group responded to Chinese script, which could be due to the differences in the phonological and morphological characteristics of the two writing systems. Additionally, the lack of an L1 advantage for the Chinese script among the Chinese participants could be due to the additional motor-gesture decoding required for Chinese characters [39,40].

### 4.2. Chinese and Korean Disyllabic Words Both Elicit N200

The results of the current study showed some differences from those reported by Zhang et al. [1]. In the current study, all three subject groups showed N200 responses to both Chinese and Korean words regardless of their Chinese language experience. This suggests that the N200 response related to orthographic processing is not an all-or-none response. Similar findings were reported in studies on alphabetic word reading, where native English speakers showed similar N170 responses to words, nonwords, and pseudo-fonts regardless of their familiarity or experience [16,24,51]. The results suggest that once expertise for a specific type of orthography is acquired, similar-looking orthography tends to elicit similar ERP responses.

In a recent study on visual-perception training, the orthography-linked centro-parietal N200 response was elicited with trained stimulus sets with conjunctive square-shaped visual objects arranged in the orthographic fashion of Chinese/Korean disyllabic compound words [52]. Taken together, these results suggest that the N200 response reflects a learned visual form response for specifically processing the conjunctive spatial structures that resemble the Chinese/Korean character configuration and alignment. It raises the question of whether different amounts of learning experience can shape the orthography-linked N200 response differently, which has not been fully addressed in previous studies on Chinese/Korean visual word recognition.

### 4.3. Subjects with Chinese Learning Experience Consistently Show N200 Enhancement for Chinese Words Relative to Korean Words

One major finding from our study is that Chinese language experience had an enhancing effect on the N200 response to Chinese script relative to Korean script in both the L1-Chinese and L2-Chinese groups. This was demonstrated through both the adaptive peak amplitude analysis and the cluster-based permutation test. As both groups had substantial experience with Chinese orthography but no prior experience with Korean orthography, their N200 responses to Chinese script were stronger than their responses to Korean script. Similar results were reported in a study by Wong and Gauthier [24] who found that L1-English speakers without any Chinese language experience had a stronger N170 response for individual Roman letters (e.g., ‘H’) than that for individual Chinese characters (e.g., ‘又’) and pseudo-fonts that resembled Korean script (e.g., ‘
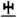
’). However, L1-Chinese–L2 English learners showed an N170 enhancement for both Chinese characters and Roman letters relative to the pseudo-font characters as they had experience with both types of orthography. The latency difference between N170 found in Wong et al. [24] and N200 found in the current study is attributable to the differences in stimuli. In Wong et al. [24], single letters, pseudo-font characters, and monosyllabic Chinese words were used as the stimuli, where in the current study, disyllabic Chinese words were used. The N200 enhancement observed in our study for Chinese disyllabic words compared to Korean in the L1-Chinese and L1-English–L2-Chinese groups suggests that language experience can modulate ERP responses related to the level of depth in orthography processing.

There are several theories on how the language-specific characteristics of Chinese characters affect orthographic processing and learning. The first relates to their visual complexity and component awareness; each Chinese character is morpho-syllabic and lacks the phonological transparency found in alphabetic–phonemic languages. More than 80% of Chinese characters are compound characters with a semantic radical component and a phonetic radical component [53]. Recognizing words in Chinese involves a holistic processing strategy at the word level rather than an analytic approach at the character level [54]. Secondly, Chinese has a high prevalence of homophones with one syllable corresponding to an average of five homophones, making radical awareness critical for vocabulary learning [55]. For instance, morphologically unrelated characters, 是 (yes), 事 (event), 视 (inspect), 市 (market), and 世 (world), all share the same syllable *shi4* with the same falling tone. Lastly, learning to write Chinese characters involves motor-gesture embodiment, and this practice modulates the centro-parietal N200 component in the brain. In a recent ERP study, Yin et al. [39] found that both writing direction and character repetition modulated the centro-parietal N200 component. These writing-direction effects were specific to Chinese characters, whereas they did not apply to Korean orthography, which are visually similar to Chinese characters but unfamiliar to the participants. The experience of learning Chinese might establish a motor-gesture decoding system for reading, which works in conjunction with the visual analysis decoding system to achieve deep orthographic processing.

A study conducted by Liu, Perfetti, and Wang [29] found that L1-English–L2-Chinese learners did not exhibit a difference in N200 response between Chinese and English words during their first semester of Chinese study. However, they showed a P200 change for Chinese words from their first to second semester. The results suggest that, with a certain amount of L2-Chinese learning experience, the N200 response to Chinese words can reach a level comparable to that of L1-English words. Despite the complexity of the Chinese writing system, beginner L1-English–L2-Chinese learners are able to quickly learn the orthography and develop analysis skills for identifying legal and illegal radical forms and positions. Wang, Perfetti, and Liu [56] found that beginner L1-English–L2-Chinese learners were sensitive to the structural composition of Chinese characters and were able to identify legal and illegal radical forms as well as legal and illegal positions of radical components. The study also showed that the L2-Chinese learners responded faster to simple characters than compound characters in a (non)-real character decision task, indicating that learners acquired the orthographic analysis skill rather quickly. The result suggested that the processing of Chinese orthography analysis was influenced by the learning experience. However, the extent to which the amount of language learning experience affects automaticity and depth of orthography analysis is still unclear as other factors like handwriting skill can also affect online orthography processing (Yin et al., 2020). In the current study, it was found that the N200 enhancement for Chinese script relative to Korean script occurred later (approximately 252 ms) for the L2-Chinese group compared to the L1-Chinese group (224 ms), indicating a latency difference between the two groups even with advanced Chinese proficiency, which is consistent with their behavioral reaction time data in judging the language script.

### 4.4. No Consistent Evidence for Language-Specific N200 Enhancement in the L1-Korean Subjects

The current study made another interesting discovery that the L1-Korean group did not show the same N200 enhancement effect in the adaptive window analysis as the other two groups, but the permutation test on point-to-point differences still indicated an enhancement effect with the Chinese script relative to the Korean script. The absence of N200 enhancement for the Korean script among the L1-Korean group in the adaptive window analysis suggested that the square-shape writing units of Chinese orthography and the complexity of spatial stroke configuration may have triggered similar N200 responses as reading the Korean script. One possibility is that, although the Korean speakers we recruited in the study might not have undergone formal instruction, they could still have encountered Chinese characters more regularly than expected due to historical, cultural, and commercial influences. When the time-point-to-time-point permutation test was used to compare the global field power (GFP) across all regions of the scalp between Chinese and Korean scripts in the L1-Korean group, the N200 enhancement effect for the Chinese script appeared at the group level. The visual distinctiveness between any two Chinese characters varies widely due to their complex internal structure, which presents a challenge in visual discrimination, and this supports the claim about the special relationship between the N200 response and the uniqueness of Chinese orthography made by Zhang et al. [1]. Each Hangul syllable is built of two to four symbols that in various combinations represent each of 24 phonemes. Chinese characters, by contrast, are composed from eight basic strokes that are combined, according to certain positional constraints, to form more than 500 component radicals [57]. Radical components are combined according to certain positional constraints to form characters. The visual distinctiveness between any two characters, thus, varies widely, and the set of characters as a whole presents a challenge in visual discrimination [58].

Although we did not observe an N200 enhancement effect for Korean words relative to Chinese words in the L1-Korean group, we found evidence of differences in peak latency between the L1-Korean group and the L1-Chinese and L2-Chinese groups. Specifically, the average peak latency for the L1-Korean group was approximately 269 ms, which was later than the other two groups. This delay may be due to the phonetic transparency of the Korean Hangul characters and simultaneous processing of orthographic and phonological information in Korean script reading. For instance, Kwon et al. [59] reported that, due to the transparency of grapheme-to-phoneme mapping in the Korean writing system, L1-Korean speakers showed an increased N250 when there was a grapheme–phoneme mismatch in a specific position in a syllable, indicating an early processing of both orthographical and phonological information in reading Korean script. The L1-Korean group’s peak latency found in our current study matched what Kwon et al. [59] found. The automaticity of converting graphemes to phonemes in Korean script reading may also contribute to the delayed N200 peak latency for the L1-Korean group. Our N200 latency data are consistent with several other reports, which directly labeled this ERP component N250 for orthographic processing of disyllabic Chinese words [60,61,62,63]. Additionally, since the L1-Korean group did not have much handwriting experience with Chinese script, the lack of motor experience may have slowed down their processing of Chinese script [39,40]. These factors together with the unique configuration of Chinese orthography, Korean language experience, and the early grapheme-to-phoneme conversion that occurred in the L1-Korean group, may have contributed to the discrepancies in the adaptive vs. fixed window analysis, making the ERP results more challenging to interpret than those of the L1-Chinese and L2-Chinese groups.

### 4.5. Limitations

The current pilot study has a limitation in that the sample size is relatively small with 10 subjects in each group [64,65]. For this reason, we consider the current report preliminary. To confirm the initial findings and explore how different levels of learning experience, including handwriting training with Chinese and Korean learning, affect the N200 response, larger samples would be necessary to achieve stronger statistical power. Additionally, it is important to note that the N200 peaks were close to the zero line, which can create challenges for statistical analysis and interpretation in terms of signal-to-noise ratio. To gain a more complete understanding of how language experience influences the orthography-specific N200 response and how it may differ across writing systems [41,42], it would be interesting to compare L1-Chinese–L2 Korean learners and L1-Korean–L2-Chinese learners to examine the mutual interlanguage influences on the N200 in addition to the L1-Chinese and L1-Korean groups.

## 5. Conclusions

In summary, the current pilot study addressed two research questions. The first research question is whether language experience with Chinese and Korean scripts will affect orthography processing of disyllabic words in the two languages in terms of behavioral performance and neural responses in real time reading. The second research question is whether the ERP component N200 is uniquely associated with Chinese orthography as claimed by Zhang et al. [1]. The results confirm significant effects of language experience on processing disyllabic Chinese words relative to visually similar Korean words. The ERP data do not support the theory that the N200 is specific or unique to Chinese orthography. Both the L1-Chinese and L1-English–L2-Chinese groups showed consistent N200 enhancement when reading Chinese words relative to Korean words regardless of whether adaptive or fixed-window ERP analysis was used. However, this N200 enhancement was not observed for Korean script relative to Chinese script in the L1-Korean group. The asymmetry of the effect of language experience on N200 among the L1-Chinese and L1-Korean groups suggested that N200 can be modulated by language experience and the unique visual-space feature of Chinese orthography at the same time. However, a cautionary note is needed here. If we assume that the L1-Korean group indeed had no prior experience with Chinese orthography, as we intended to control, then the absence of a distinct N200 difference between Korean and Chinese scripts suggests that specific visual features unique to Chinese orthography (like the stroke patterns and arrangement of two square shape blocks) can trigger a relatively strong N200 response regardless of learning experience. However, it is difficult to rule out the fact that many participants within the L1-Korean group had incidental exposure to Chinese characters due to cultural influences given the historical use of Chinese characters in Korean writing. Overall, our preliminary findings suggest that the N200 amplitude and latency reflect the depth of early visual processing of disyllabic square-shaped writing units (such as Chinese and Korean scripts), which is influenced by language-specific orthographic properties and language learning experience. While limited in sample size for an ERP study, the results can inform and inspire further cross-linguistic investigation on visual word reading with implications for language education.

## Figures and Tables

**Table 1:** Demographic information of participants (N = 5 females and 5 males for each group).

	L1-Chinese Group		L1-Korean Group		L1-English L2-Chinese Group	
	Mean (sd)	Range	Mean (sd)	Range	Mean (sd)	Range
Age at experiment	21 (1.3)	19–23	21 (1.2)	18–24	22 (1.4)	21–23
Years learning Chinese	native		0	0	4 (2.3)	4–7
Years in immersion in China	native		0	0	0.5 (1.8)	0–0.8
Chinese language experience	native		none		advanced	
Korean language experience	none		native		none	

## Data Availability

Data reported in this study are available from the corresponding author upon reasonable request due to resource restrictions and ethical considerations.
